# Spine alignment in men during lateral sleep position: experimental study and modeling

**DOI:** 10.1186/1475-925X-10-103

**Published:** 2011-11-30

**Authors:** Karim Leilnahari, Nasser Fatouraee, Mahmoud Khodalotfi, Mohammad Amin Sadeghein, Yekta Amin Kashani

**Affiliations:** 1 Department of Biomedical Engineering, Science and Research Branch, Islamic Azad University, Tehran, Iran; 2 Department of the Biomechanical Engineering, Faculty of Biomedical Eng., Amirkabir University of Technology (Tehran Polytechnic), Tehran, Iran

**Keywords:** Bed design, Spinal alignment, sleep Posture, custom-made arrangement, mattress stiffness, sleep system

## Abstract

**Background:**

A proper sleep system can affect the spine support in neutral position. Most of the previous studies in scientific literature have focused on the effects of customary mattresses on the spinal alignment. To keep the spine in optimal alignment, one can use sleep surfaces with different zonal elasticity, the so called custom-made arrangements. The required stiffness of a sleep surface for each individual can be obtained by changing this arrangement applying the experimental method and modeling.

**Methods:**

In experimental part, the coordinate positions of the markers mounted on the spinous processes of the vertebrae of 25 male volunteers were registered in frontal plane through the optical tracking method and so the spinal alignment was obtained in lateral sleep position on soft and firm surfaces and on the best custom-made arrangement. Thereupon the π-P_8 _angles were extracted from these alignments and then were compared with each other. In modeling part the anthropometric data of four different types of volunteers were used. And then the models built in BRG.LifeMOD (ver. 2007, Biomechanics Research Group, Inc., USA) based on these data and in accordance with the experimental tests, were analyzed.

**Results:**

The one way ANOVA statistical model and the post hoc tests showed a significant difference in the π-P_8 _angles between soft & custom-made and soft & firm mattresses at the p = 0.001 level and between firm & soft mattresses at the p = 0.05 level. In modeling part, the required stiffness of the sleep surface for four weight-dimensional groups was acquired quantitatively.

**Conclusions:**

The mattress with a custom-made arrangement is a more appropriate choice for heavier men with pronounced body contour. After data fitting, it was observed that the variations of spinal alignment obtained from both methods have the same trend. Observing the amount of required stiffness obtained for the sleep surface, can have a significant effect on keeping the spine healthy.

## 1. Introduction

Human being has no active control on spine alignment during sleep. A proper sleep system can align the spine to some extent in its neutral posture which is the same as the spine alignment in upright position [1]. A non-neutral posture can apply lateral bending and unbalanced loading on intervertebral disks and facet joints. Growth and restoration of intervertebral disks as a hydrated soft tissue are based on the amount of pressure and its manner of application on them [2]. With the change of the direction of gravity vector during sleep, the intervertebral disks are unloaded and can rehydrate to restore their elasticity.

Various researches have been done on the effect of mechanical parameters of sleep system (cushion, mattress, bed base) on the posture of individual, manner of load distribution, decubitus ulcer, temperature and heat transfer, comfort, and physiological responses of the body like muscle relaxation. The effect of these parameters on the soundness of vertebral column has been also investigated. Soft surfaces cause the increase in low back pain due to incorrect support of vertebral column [3-12]. Thereagainst, mattresses with high stiffness lead to shoulder pain, and cause the decrease in sleep quality and improper distribution of body loads [13-15].

Kovacs et al. observed that patients with low back pain feel less comfortable on a mattress with high stiffness than on a mattress with average stiffness [16]. Jacobsen et al. showed that using an individualized sleep system, in comparison with the sleep system which an individual usually uses, causes the shoulder and back pain of the individual to decrease and his sleep quality to increase [13]. It has been found in another study that sleeping on a firm surface leads to C-form curvature of spine in a frontal view [2]. De Vochta et al. showed also in their study some differences in maximum pressure parameter which was related directly to the weight of the individuals [17].

Haex et al. dealt with the basic modeling of spine, sleep system and individual and found out that there is possible to make the sleep system appropriate for the individual needs [1]. Gefen et al. could calculate the effect of the back support angle on deep muscles stresses through the finite elements method [18].

One suggestion to maintain the spine in optimal alignment during sleep is the use of surfaces with different elastic properties, the so called custom-made arrangement, and classification of individuals based on gender, anthropometry, body weight distribution and sleeping habits. However, individuals with different sleeping characteristics require different zonal stiffnesses in mattress in order that the mattress maintains their spine in natural alignment during sleep. Every person should have an individually adapted sleep system according to his physical needs and conditions.

The first part of this research was dedicated to the study of the effect of body dimensions, gender and weight in men during lateral sleep position on soft and firm mattresses and to obtaining a custom-made arrangement. The major problem of empirical methods is their performance since many measurements are needed to establish an exact relationship between human physical characteristics and the characteristics of an optimal sleep system for all groups of individuals. Moreover, finding adequate number of subjects for tests and collecting different sleep systems are often difficult. In order to overcome these complexities, one can study different sleep systems through modeling.

In the second part, the modeling of individual and his spine was used to find out the individual needs during lateral sleep position, and finally the results of both parts were compared to each other.

In previous studies, the required stiffness for every person in different parts of his body on a mattress with variable zonal stiffnesses to maintain the spine in correct alignment, has not been investigated quantitatively and most studies have focused on the effect of customary mattresses on the spine alignment. But in the present study, after extraction of the spine alignment in men in lateral sleep position by experimental tests, the precise modeling of the sleep system and the individual was performed as the innovation of the investigation. Thereafter, the weighting factors according to the findings of experimental tests were assigned to the modeling results and finally the required stiffness for each body-weighting group was extracted quantitatively.

## 2. Materials and methods

### 2.1. Experimental testing

#### 2.1.1. Test level

Rigid base was used for all mattresses, because the stiffness change in mattress base can alter the results completely. Polyurethane foam and a layer of memory foam were used for soft surface. The calculated value of E/C for the set was 2476 mm^2 ^and this value belongs, in accordance with the standard definition of LGA, to the class of soft surfaces [14]. For choosing a firm surface, a very firm surface was preferred in order to obtain better outcomes.

According to LGA standards, E (N.mm) is the required work for compressing the core with a force which maximally equals to 450 N and C (N/mm) is the average differential stiffness. E can be obtained by calculation of the total area under the loading curve in between 0 and 450 N limits and C is obtained by calculation of average slope of tangents to loading curve at points of 210, 275 and 340 N [1].

When building a mattress with elements of different stiffness, a simultaneous combination of polyurethane and spiral pressure springs with different wire diameters for static loading was selected. The behavior of each foam and spring element was studied using a pressure test under constatnt velocity of 2 mm/min with a Zwick/Roell device and in accordance with the standard protocol ISO 2430; 2001 [19].

#### 2.1.2. Anthropometric measurments and marker attachment

After a primary interview, 25 volunteers from male students of University of Sciences and Researches, who were in the height range of 175 ± 5 cm, were invited for co-operation. After being examined to ensure that they do not suffer any spinal deformations, the volunteers signed the consent form of tests. The personal information and 18 anthropometric parameters of volunteers were obtained by an anthropometry specialist. Dimensions, girth, height and depth of thorax, shoulder, belly and pelvis of volunteers were measured. The research assistant determined the location of 12 spinous processes of vertebrae C_7_, T_1_, T_2_, T_3_, T_4_, T_6_, T_8_, T_10_, T_12_, L_1_, L_2 _and L_5 _of each volunteer, when sleeping prone on mattress, through a similar process of spine palpation [20] and attached a marker on each spinous process. Four active markers were set as the reference markers, two of them were placed in a distance of 5 cm from the fifth lumbar vertebra (L_5_) and the other two in a distance of 10 cm away from the last cervical vertebra (C_7_). All Experiments performed on volunteers has been done with respect of Helsinki declaring. This procedure is approved by medical ethics committee of Kurdistan University of Medical Sciences. The final approval letter reference No. is 17926/4021.

#### 2.1.3. Image capturing

After reviewing the different methods for extraction of the spinal alignment, the external measurement technique of optical tracking was selected. For this aim, two digital cameras (model: DCR-TRV356E, Sony Corporation, Tokyo, Japan) were used. One of the cameras was installed in a distance of 4 m from the mattress frame plate and at the same height of the initial mattress surface. The volunteers and the camera were always set at a fixed position so that the camera could capture both the back of the volunteer and the reference coordination and calibration set. Another camera with the same specifications was installed on the ceiling of laboratory perpendicular to the mattress surface. The images of both cameras were simultaneously observed and saved during the tests.

#### 2.1.4. The posture of individual

The volunteer was asked to lie in a lateral position so that his back surface is perpendicular to the mattress surface and is completely in the capturing frame of the camera. In case of any change in position by individual leading to the displacement of his back skin with respect to the spinal column, the research assistant brought the markers back to the location of spinous processes.

In order to prevent the twist of spinal column, the volunteer was asked to turn himself a little so that the reference markers align as much as possible in the set alignment both in Sagittal and frontal planes and the whole process was controlled through the images of top camera. To prevent the pillow from affecting the alignment of cervical part of the spine, the height under the head of each person was so set that the assumed line passing through the cervical vertebrae is horizontal. Using the palpation method, the trunk angle - i. e. the angle between the lateral midline of trunk and femur - and the flexion angle of knee were set respectively to 135 and 90 degrees. These angles are, according to previous studies, the angles of neutral position of these limbs [21]. The coordination related to the location of markers and spinous processes were extracted. The software took the image distortion and image depth errors into account through data obtained from the top camera and minimized the selection error by finding the locus of maker center.

#### 2.1.5. Testing on soft, firm and custom-made surfaces

Each volunteer was asked to lie in lateral sleep position according to research protocol, two times on a soft surface and two times on a firm surface, and each time the necessary images were captured. In case that the initial combination of sleep system, after lying of the individual on it and extraction of his spinal alignment, couldn't bring the spinal alignment in saggital plane close to the optimal alignment, the process was repeated several times by modification of the combination arrangement so that the spine in lateral sleep position is aligned in optimal alignment. Finally, the last arrangement was selected as the optimal arrangement for the individual.

In accordance with the definition of optimal spinal alignment, a frontal projection of the spinal column has to approximate the reference shape in normal people, which is a straight line. The deviation from this line is quantified by P parameters [1]. The spinal slope often shows a discontinuity around vertebra T_11 _at the transition from the flexible lumbar area to the more rigid thoracic area, and therefore the measurement points are subdivided into two parts separated by this vertebra. The angle between these two lines defines the frontal parameter P_8_. In quantitative and statistical investigation of performance of a custom-made mattress in lateral sleep position, the angle π-P_8 _was used as the optimization criterion. For the precise investigation of spinal column, all the alignments have to be of the same scale. The MATLAB software was used to match them in the same scale and to determine the mapping parameters.

## 3. Modeling

The BRG.LifeMod software version 2007 was used to build a model and to do the analysis. According to scientific research literature, the men in whom the proportion of shoulder width (the distance of two deltoid muscles) to their pelvis width is above 1.45 are termed as triangular and the ones with a proportion under 1.45 are described as square [1]. The anthropometric characteristics of some volunteers who were triangular and square in accordance with the definition, were input into data bank and the models of four men were named briefly as follows: the Heavy Triangular Man (HTM) with the height of 181 cm and the weight of 103 kg and the proportion of 1.59, the Heavy Square Man (HSM) with the height of 183 cm and the weight of 93 kg and the proportion of 1.33, the Light Triangular Man (LTM) with the height of 182 cm and the weight of 74 kg and the proportion of 1.52, the Light Square Man (LSM) with the height of 184 cm and the weight of 76 kg and the proportion of 1.28.

The software subdivides the spinal column as default into cervical, thoracic and lumbar parts and articulates only these three parts by joints. Regarding the precision required in studying the spinal alignment, the remodeling of vertebrae has been carried out to make the definition of joints between the vertebrae possible.

For all body joints, except for intervertebral disks, the standard hybrid III [1] values were used. To create the desired stiffness and range of motion (ROM) for intervertebral disks joints, the joint damping coefficient and the natural ROM of the joint were input [22]. The body posture in model was applied according to the protocol used in experimental test.

The cubic volumes in MSC ADAMS 2005 were used to model the mattress with a custom-made arrangement. Blocks with dimensions of 20 × 100 × 10 cm (height × width × length) were designed. No joint was provided between the blocks so that they could move freely and independently. Thereafter, the built models were placed on the surface of these volumes. Contact was produced between the body limbs and the blocks. The properties related to damping and friction coefficient, were in all cases constant.

Each of the four guide models was first laid on the soft and the firm surface and the analyses were carried out. Reaching to a steady state, the location of vertebrae mass centers were extracted for each model on every surface. Changing the blocks stiffness, it was tried to bring the spinal alignment in frontal plane as close as possible to the neutral position. Changing the stiffness parameter of a surface led in some cases to the alteration of spinal alignment in other areas. The changes were made several times to find the required zonal stiffness of the surface for each body part of the model.

## 4. Findings

### 4.1. Experimental findings

**Figure 1:**
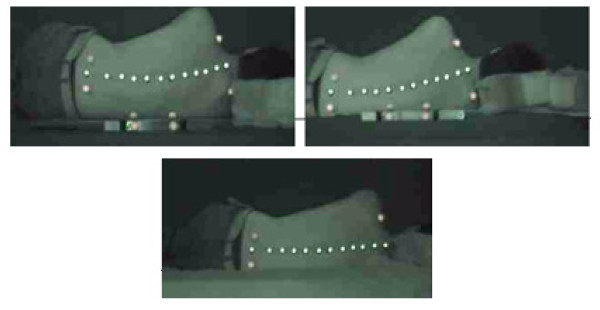
**Images of a volunteer**. Images of a volunteer captured on custom-made (down), soft (up & right) and firm (up & left) surfaces.

For all subjects, the spinal alignments on soft and firm surfaces and on the last surface with costum-made arrangement were extracted. Figure 1 shows one of the volunteers on soft, firm and custom-made surfaces.

**Figure 2:**
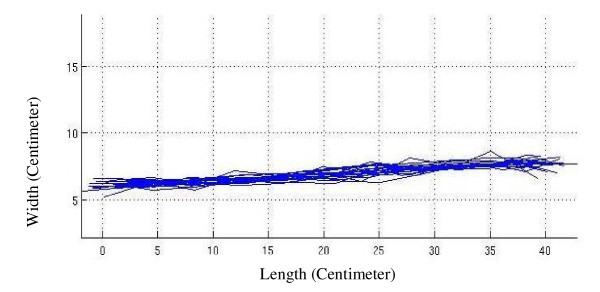
**The spinal alignment graph**. The alignment graph of spinal midline of volunteers after matching the curves in-between vertebra L_5 _and vertebra C_7 _on the surface with a custom-made arrangement.

**Figure 3:**
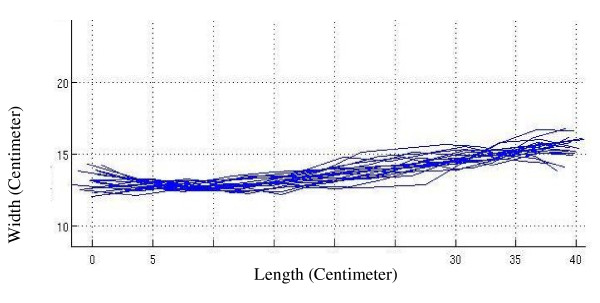
**The spinal alignment graph**. The alignment graph of spinal midline of volunteers after matching the curves in-between vertebra L_5 _and vertebra C_7 _on the firm surface.

**Figure 4:**
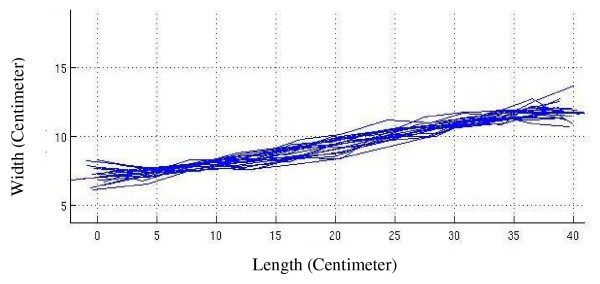
**The spinal alignment graph**. The alignment graph of spinal midline of volunteers after matching the curves in-between vertebra L_5 _and vertebra C_7 _on the soft surface.

In Figure 2 the alignment graph of spinal midline (passing through the spinous processes) of volunteers is shown after matching the curves in-between vertebra L_5 _and vertebra C_7 _on the surface with a custom-made arrangement. Figure 3 shows the same graph on the firm surface and Figure 4 shows it on the soft surface.

Investigating the descriptive statistics of data using SPSS version 11.5 revealed that data distribution was normal and symmetric. Hence, the parametric models were used.

**Figure 5:**
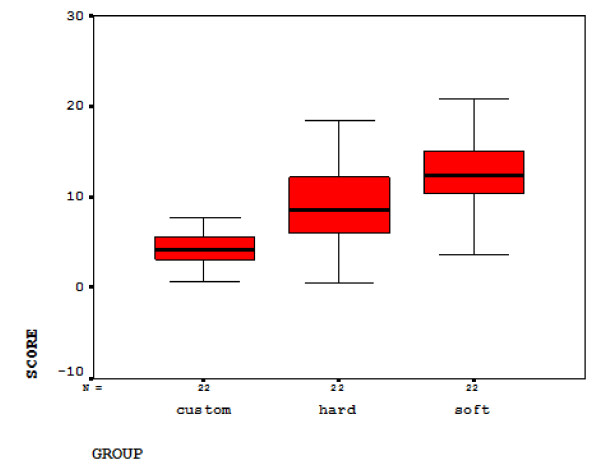
**graph of π-P_8_**. The dispersion graph of π-P_8 _values in three firm, soft and custom-made surfaces.

Figure 5 shows the dispersion of data relating to the soft, firm and custom-made surfaces. The smallest amount of π-P_8 _among these groups belonged to the custom-made surface (4.10°) and after it stood the firm surface (8.9°) and the soft surface (12.66°).

The angle π-P_8 _was defined as the dependent variable and the mattress types (soft, firm and custom-made mattresses) were considered as the independent variable. So the statistical model of one way ANOVA modified by Welch was used for examination. The statistical results showed that F = 29.712 > 3 after applying the Welch modification, F_w _= 46.31 > 3 is meaningful at the level p = 0.001 < 0. 05.

The Levene F value (5.77) is also statistically meaningful, and it is therefore necessary to use post hoc tests related to heterogeneous variances, like the test of Tomhans. As the results of Tomhans' post hoc test for the three mattress types show, there are meaningful differences between soft-custom-made and soft-firm mattresses at the level p = 0.001 and soft-firm mattresses at the level of p = 0.05.

### 4.2. Modeling findings

**Figure 6:**
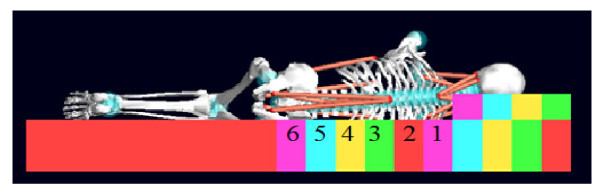
**Model on the mattress**. The frontal projection of HTM model in lateral sleep position on the custom-made surface, along with the numbers of the blocks in contact with body.

Figure 6 shows a projection of HTM model on the last custom-made surface. Table 1 presents the normalized spring stiffness value necessary for men in accordance with the zones specified in Figure 6.

**Table 1:** The normalized spring stiffness value in accordance with the zones specified in figure 6

Model Name	Body Shape	Zone number					
		
		1	2	3	4	5	6
HTM		66.2	66.2	88.2	88.2	82.4	82.4

HSM		58.8	58.8	76.5	76.5	100	100

LTM		50.0	55.9	58.8	58.8	52.9	52.9

LSM		55.9	55.9	58.8	58.8	58.8	58.8

### 4.3. Comparring the modeling and experimental test findings

**Figure 7:**
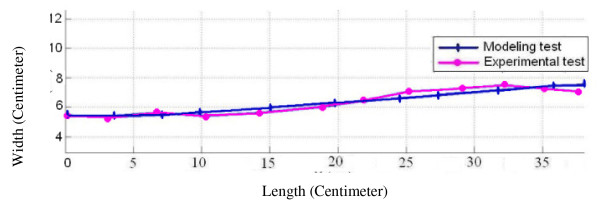
**The spinal alignment graph**. The spinal alignment graphs of LTM in-between vertebra L_5 _and vertebra C_7 _on the soft surface, obtained through both modeling and experimental method.

**Figure 8:**
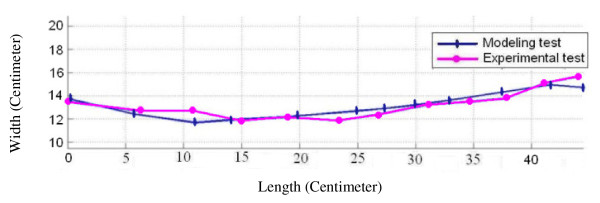
**The spinal alignment graph**. The spinal alignment graphs of LSM in-between vertebra L_5 _and vertebra C_7 _on the firm surface, obtained through both modeling and experimental method.

After obtaining the anthropometric data of volunteers as mentioned before, the distinct examples of each HTM, HSM, LTM, LSM group were determined and an exact model was built in software using these anthropometric data. Below, the spinal alignment graphs of distinct volunteers obtained through both modeling and experimental method have been shown as a sample (Figure 7 &8).

## 5. Discussion

Due to the complex structure of disks, the axial twist of intervertebral disks is practically up to about 6 degrees tolerable in each disk; the increase in twist raises the nucleus pressure [23]. The incorrect sleep position causes improper bending in intervertebral disks. The prolonged repetition of unnatural stresses brings the tissue in damage phase. The forced change in sleep position of people having the awakening signs, could improve their sleep quality [21].

The curvature of body contour in frontal plane has no linear relationship with body weight distribution. The human body is wider as well as heavier in pelvic area. This, however, is not in the thoracic area the case; the body in this region is due to presence of lungs wider but not heavier. Therefore, there is an incorrect spinal support when people lie in a natural lateral sleep position on a very firm mattress, as only the wide areas of body are supported, i. e. just the shoulder and pelvis receive good support.

If the shoulders can't sag into the mattress, then the support of neck and back won't be satisfactory and the shoulder joint will suffer in contact surface from high stresses leading to pain and joint stiffness. For male subjects regardless of their body shape and weight, the existence of a soft shoulder zone will improve the spinal alignment and is therefore necessary.

As it is seen in Figure 2 which is related to a custom-made surface, an arrangement with different stiffness zones has maintained the natural alignment in a frontal plane. In Figure 3, the spinal column on a firm mattress has bent down in the lumbar area. This produces a lateral bending, as only shoulder and pelvis receive good support on such surfaces.

While sleeping, human takes a posture between lateral and prone sleep positions to remove this harmful lateral bending. This posture is called the three dimensional posture and most of the people take it during sleep on very firm surfaces.

In contrast, the softness of the mattress in Figure 4 has caused the pelvic area to sag more into the mattress, while this is not the case for the upper part of the body (with a relatively lower mass). As a result, the position of the vertebra C_7 _on soft surfaces is higher than that of pelvis.

During the investigation of results obtained from statistical examinations, it was observed that the mattress with a custom-made arrangement could minimize the average π-P_8 _angle in frontal plane in lateral sleep position, and in fact, the custom-made mattress, compared to soft and firm surfaces, could bring the spinal alignment in frontal plane close to the neutral alignment. The average value of π-P_8_, as it is seen in Figure 5, decreases from soft, and then firm, to custom-made surfaces.

In comparing the soft and firm surfaces, the statistical results introduced the firm mattress as a more proper choice for lateral sleep position. This point corresponds with the former findings of researchers. In fact, a firm mattress prevents the deflection of spine in pelvic area in lateral sleep position and causes the spine to align in frontal plane better than a soft surface. That is why most of the people choose the firm surface over the soft one to reduce the low back pain.

Table 1 presents the stiffness of each mattress zone for a person regarding the gender and anthropometric characteristics. To provide the possibility of comparison, the stiffness values in this table have been normalized with respect to the maximum stiffness value. In HTM, the stiffness of shoulder area on zones 1 & 2 is obviously different from the stiffness of other zones. The greater stiffness of zones 3 & 4 in comparison with zones 5 & 6 in heavy triangular men is aimed to prevent the deflection of inward curved contour of the waist area in frontal plane. It means that the spine in this area has been supported completely by preventing the spine deflection in this zone and restricting the deflection in zones 5 & 6.

As it is seen, the heavy people principally benefit more than the light people from a custom-made system in lateral sleep position. Furthermore, the triangular subjects benefit, as it becomes manifest from the stiffness values, more than the square people from a custom-made system in lateral sleep position.

To validate the modeling method, as mentioned in section "methods", the anthropometric characteristics of some distinct volunteers in four groups of HTM, HSM, LTM and LSM, were input into software and the resulted spinal alignment of volunteers from the experimental data were drawn in a diagram. The Figures 7, 8 show a comparison between the experimental method of obtaining the spinal alignment and the modeling method. The modeling method, to a great extent, could predict the spinal alignment of different people on soft and firm surfaces.

## 6. Conclusion

Measurements show that a too soft or too firm mattress is no good choice for most of men. A mattress with a custom-made arrangement is proper for all the people, especially the men with a heavy weight and the ones who have a more pronounced contour. Such studies are applicable in field of industrial production of new sleep systems in order to better observe the ergonomic factors and to keep the spine healthy.

Using a custom-made mattress and sleep system is a proper way to better support of spine during sleep. By subdividing people into different, but restricted, weight and dimensions groups, designing and building mattresses with a proper arrangement for those specific groups become possible.

It was found in this research that modeling is a proper tool to predict the spine behavior on a sleep system, as it could predict the spine behavior to great extent. Through the modeling method, one can predict the mechanical behavior of materials and suggest them to the sleep system industry.

The following projects are suggested for future investigations; the prediction of custom-made arrangements for people without carrying out the tests, investigating the effects of mattress stiffness in supine sleep position, and modeling of the people who had an operation on their spine.

## Competing interests

The authors declare that they have no competing interests.

## Authors' contributions

All Authors have been involved in data aquisation, data Analysis, modeling, drafting and revising manuscript. All authors read and approved the final manuscript.

## Authors' information

Karim Leilnahari is a member of Biomechanics Department in Biomedical Engineering Faculty at Science and Research Branch of Islamic Azad University. He received his B.Sc. in Mechanical Engineering from K. N. TOOSI University of Technology in Tehran and his M.Sc. and Ph.D. in field of Biomedical Engineering from Science and Research Branch of Islamic Azad University, Tehran.

Nasser Fatouraee is associate professor and the head of Biomechanics and Sports Engineering Departments in Biomedical Engineering Faculty at Amirkabir University of Technology. He received his B.Sc. in Mechanical Engineering from Sharif University of Technology in Tehran, Iran, and his M.S. and Ph.D. in Mechanical Engineering from Laval University in Quebec, Canada. He did his postdoctoral studies at Washington University Medical Center.

Mahmoud Khodalotfi received his B.Sc. in Biomedical Engineering in 2010 from Science and Research Branch of Islamic Azad University, Tehran.

Mohammad Amin Sadeghein studies his master in Biomedical Engineering in the school of Engineering at Bridgeport University. He received his B.S. in Biomechanical Engineering from Science and Research Branch, Islamic Azad University, Tehran, Iran in 2010

Yekta Amin Kashani received her B.Sc. and M.Sc. in Biomedical Engineering from Science and Research Branch of Islamic Azad University, Tehran.
